# In vivo therapeutic efficacy and pharmacokinetics of colistin sulfate in an experimental model of enterotoxigenic *Escherichia coli* infection in weaned pigs

**DOI:** 10.1186/s13567-016-0344-y

**Published:** 2016-05-27

**Authors:** Mohamed Rhouma, Francis Beaudry, William Thériault, Nadia Bergeron, Guy Beauchamp, Sylvette Laurent-Lewandowski, John Morris Fairbrother, Ann Letellier

**Affiliations:** Chaire de recherche en salubrité des viandes (CRSV), Faculté de médecine vétérinaire, Université de Montréal, Saint-Hyacinthe, QC J2S 7C6 Canada; Groupe de recherche et d’enseignement en salubrité alimentaire (GRESA), Faculté de médecine vétérinaire, Université de Montréal, Saint-Hyacinthe, QC J2S 7C6 Canada; Centre de recherche en infectiologie porcine et avicole (CRIPA), Faculté de médecine vétérinaire, Université de Montréal, Saint-Hyacinthe, QC J2S 7C6 Canada; Groupe de recherche en pharmacologie animale du Québec (GREPAQ), Faculté de médecine vétérinaire, Université de Montréal, Saint-Hyacinthe, QC J2S 7C6 Canada; Groupe de recherche en épidémiologie des zoonoses et santé publique, Faculté de médecine vétérinaire, Université de Montréal, 3200 rue Sicotte, Saint-Hyacinthe, QC J2S 7C6 Canada; OIE Reference Laboratory for Escherichia coli, Faculté de médecine vétérinaire, Université de Montréal, Saint-Hyacinthe, QC J2S 7C6 Canada

## Abstract

Enterotoxigenic *Escherichia coli* (ETEC: F4) associated with post-weaning diarrhea (PWD) in pigs has developed resistance against several antimicrobial families, leading to increased use of colistin sulfate (CS) for the treatment of this disease. The objective of this study was to determine the efficacy of oral CS treatment in experimental PWD due to ETEC: F4 challenge and determine the effect of this challenge on CS intestinal absorption. In this study, 96 pigs were divided into two trials based on CS dose (100 000 or 50 000 IU/kg). Fecal shedding of ETEC: F4, total *E. coli*, and CS-resistant *E. coli*, diarrhea scores, and weight changes were evaluated. Colistin sulfate plasma concentrations were determined by HPLC–MS/MS. Regardless of the dose, CS treatment resulted in a reduction of fecal ETEC: F4 and total *E. coli* shedding, and in diarrhea scores but only during the treatment period. However, CS treatment resulted in a slight increase in fecal shedding of CS resistant *E. coli* and did not prevent weight loss in challenged pigs. In addition, challenge with ETEC: F4 resulted in an increase of CS intestinal absorption. Our study is among the first to demonstrate that under controlled conditions, CS was effective in reducing fecal shedding of ETEC: F4 and total *E. coli* in experimental PWD. However, CS treatment was associated with a slight selection pressure on *E. coli* and did not prevent pig weight loss. Further studies are needed in field conditions, to better characterize CS therapeutic regimen efficacy and bacterial resistance dissemination.

**Electronic supplementary material:**

The online version of this article (doi:10.1186/s13567-016-0344-y) contains supplementary material, which is available to authorized users.

## Introduction

*Escherichia coli* post-weaning diarrhea (PWD) is an economically important disease in pig production worldwide [1–3]. This disease affects pigs mostly during the 2 weeks after weaning and is characterized by a reduction in feed intake, poor growth rate, diarrhea and mortality [3]. These disturbances are most commonly associated with the proliferation of enterotoxigenic F4-positive *E. coli* (ETEC: F4) [3], the most predominant sero-virotypes being O149: LT: STb: F4 and O149: LT: STa: STb: F4 [3, 5]. Small intestine epithelial cell adhesion and subsequent colonization by ETEC: F4 is mediated by the F4 fimbriae via specific receptors (F4R), crucial in determining the susceptibility of pigs to ETEC infection [3, 4]. Because ETEC: F4 isolates from PWD have shown a high frequency of resistance to multiple antimicrobials [1, 5], therapeutic failure is common and alternative molecules need to be found. Colistin sulfate (CS), a cationic antimicrobial peptide, is one possible candidate for the treatment of PWD, which is approved for use in pigs in several countries [6, 7]. However, CS is not yet approved for use in pigs in other countries such as Canada and is used under veterinarian responsibility for the treatment of PWD [8].

The bactericidal effect of CS is the result of an electrostatic interaction between the cationic elements of CS and anionic lipopolysaccharide (LPS) molecules in the membrane of Gram-negative bacteria, leading to the displacing of magnesium (Mg^2+^) and calcium (Ca^2+^)—stabilizers of LPS molecules—from the LPS [9]. This process results in an increase in the permeability of the cell envelope, leakage of cell contents, and subsequent cell death [10, 11].

Several studies from different countries have reported isolation from pigs of *E. coli* resistant to colistin [12–17]. The most common mechanisms of resistance to CS in *E. coli* are modifications of the LPS with the addition of positively charged groups, such as L-4-aminoarabinose (L-Ara4N) and/or phosphoethanolamine (pEtN) [18–20]. More recently, Liu et al. have demonstrated the presence of a stable plasmid mediated *mcr*-*1* gene that encodes for *E. coli* colistin resistance [21].

In pigs, CS is mainly administered per os, at the recommended dose of 50 000 IU/kg body weight (bw) every 12 h for a period of 3–5 consecutive days for the treatment of intestinal infections caused by Enterobacteriaceae [6, 22]. However, this dose regimen is often not respected on farms [6]. Several reports have shown that the recommended dose [23–25] or duration [23, 25] of CS treatment is often surpassed.

In addition, the efficacy of CS at the dose of 50 000 IU/kg for the clinical treatment of PWD has not been investigated and no data are available in the literature on the role of this therapeutic regimen in exacerbating of *E. coli* resistance in pigs. Several studies have confirmed that CS is poorly absorbed in pigs after oral administration [8, 22]. However, little is known of the effect of ETEC: F4 infection with clinical PWD on CS intestinal absorption, following the use of CS in a conventional therapeutic regimen. An increase of CS intestinal absorption could have an impact on the withdrawal time following oral administration of this antibiotic. Moreover, in countries where CS is approved in pig, this varies from 1 to 7 days [6].

Hence, the main objective of the present study was to evaluate the effect of CS treatment in an experimental PWD model on fecal ETEC: F4 and total *E. coli*, on *E. coli* resistance to CS, on fecal consistency, growth rates, and rectal body temperature of weaned pigs. In addition, the effect of ETEC: F4 infection on CS intestinal absorption levels was determined using a high-performance liquid chromatography coupled with tandem mass spectrometry (HPLC–MS/MS).

## Materials and methods

The experimental protocol (14-Rech-1729), was reviewed and approved by the Ethics Committee on Animal Use of the Faculty of Veterinary Medicine (FVM) of the University of Montreal, and it was performed in accordance with the guidelines of the Canadian Council on Animal Care (CCAC).

### Animals, experimental design and housing

A total of 96 Duroc-Yorkshire-Landrace pigs were used to carry out the experiment, animals were housed at a biosecurity level 2 agro-environmental platform for farm animals of the FVM.

Pigs were selected based on the presence of the F4 receptor gene by PCR–RFLP as previously described [26] at 4 days of age. Two trials of 48 pigs were conducted using different doses of CS [100 000 IU/kg (trial 1) or 50 000 IU/kg (trial 2)]. In each trial, four groups of 12 pigs were constituted: challenged treated, challenged untreated, unchallenged treated, and unchallenged untreated.

After weaning (21 days old), pigs were fed a standard non-medicated ration for post-weaning pigs and had unlimited access to feed and water throughout the 7 weeks of the study. The temperature of the room was kept at 24–26 °C. In both trials, challenged groups were placed in the same room, although each group (*n* = 12) was housed in a separate pen. The two unchallenged groups were placed in two different rooms. Each pen had a stainless-steel feeder and a low-pressure nipple drinker. In order to avoid contamination of control groups, biosecurity measures were applied, including use and changing of boots, coveralls and gloves before entering each room.

### ETEC: F4 Oral challenge and antimicrobial administration

For experimental infection of pigs, a nalidixic acid-resistant (Nal^r^) variant of ETEC: F4 strain ECL8559 (O149: LT: STa: STb: East1: paa: hemβ: F4), kindly provided by the *E. coli* Laboratory as described previously [8], was used. The strain was passaged in a weaned pig to enhance its pathogenicity. A hemolytic, Nal^r^ colony isolated from the feces of this pig was confirmed to be positive for O149 and the virulence genes F4, STa, STb, LT by multiplex PCR as previously described [27]. This strain, designated ECL8559A, was used in the experimental challenge in this study. After 1-week of acclimatization, 28-day-old pigs in the challenge groups were orally gavaged with 10^9^ CFU of the ETEC: F4 strain in 5 mL of trypticase soy broth (Difco Laboratories, Inc., Detroit, MI, USA) following the administration of 10 mL of CaCO_3_ to neutralize gastric acid.

Colistin sulfate (Bond & Beaulac Inc., QC, Canada) was administered by oral gavage in 5 mL of water using a polyethylene tube attached to a syringe, at a dose of 100 000 or 50 000 IU/kg in trials 1 and 2 respectively. CS administration was started when at least two pigs from the challenged groups showed PWD symptoms (i.e. score 2 of diarrhea, lethargy and anorexia), and continued twice a day for 5 successive days.

### Fecal sampling and microbiological analysis

Fresh fecal samples were obtained from pigs using pre-weighed sterile rectal swabs (Puritan Medical Products, Guilford, Maine, USA). Bacteriological examination of fecal samples was performed 1 day before and 1, 2, 3, 5, 6, 7, 8, 10, 13, 20, 27, 36 days after oral challenge to evaluate fecal excretion of the challenge ETEC: F4 strain and total *E. coli* count. One millilitre of buffered peptone water solution (BPW) was added to each swab and selected dilutions were plated on MacConkey agar and 5% bovine blood agar plates containing nalidixic acid at 50 μg/mL (Sigma-Aldrich Canada Ltd., Oakville, ON, Canada) to count the total *E. coli* population and the hemolytic challenge ETEC: F4 strain respectively, as previously described [8, 26]. In parallel, 5% bovine blood agar plates containing nalidixic acid at 50 μg/mL and CS at 2 μg/mL and MacConkey agar plates containing CS at 2 μg/mL were used to enumerate the CS resistant hemolytic challenge ETEC: F4 and total *E. coli* population respectively. The plates were incubated aerobically for 24 h at 37 °C. Isolates recovered from media containing 2 μg/mL of colistin were considered to be putative CS-resistant, as previously described [16]. All samples were processed on the day of collection. Rectal swabs were weighed before and after sampling of pigs for individual fecal material quantification.

The isolates on MacConkey agar were confirmed as *E. coli* by colony morphology and biochemical analysis [28]. Hemolytic colonies on blood agar were confirmed as ETEC: F4 by multiplex PCR using published primers [29–31]. The minimum inhibitory concentration (MIC) was determined as the lowest CS concentration that resulted in the inhibition of bacterial growth. The MIC was determined for the challenge strain before animal inoculation, and for confirmed *E. coli* isolates recovered from agar plates containing CS at 2 μg/mL after challenge. The MIC was carried out by microdilution method using a sterile 96-well polystyrene microplate, as previously described [8]. The MIC was only evaluated on isolates from trial 2 (50 000 IU/kg), representing the most common dosage used in PWD treatment worldwide.

At 36 days post-challenge, pigs were euthanized and necropsies were performed.

### Health status assessment

After the oral challenge, pigs were observed daily for signs of anorexia, lethargy and diarrhea. The severity of diarrhea was assessed visually by using a fecal consistency scoring (0, normal; 1, soft feces; 2, mild diarrhea; 3, semi liquid diarrhea and 4, liquid diarrhea) as described by Jamalludeen et al. [32]. The rectal body temperature was monitored daily using a digital thermometer.

Pigs were weighed individually using an electric scale prior to inoculation and at 6, 19 and 35 days after beginning CS treatment.

### Blood sampling and pharmacokinetic analysis

Blood samples (3 mL) were collected using potassium EDTA tubes, from the jugular vein of eight pigs in each treated group, challenged or not of the two trials, at 0.5, 12, 24 and 48 h after the last CS oral administration on day 5.

Plasma was separated by centrifugation at 3000 *g* for 10 min and stored at −20 °C prior to analysis. These samples were used to determine CS plasma concentrations by high performance HPLC–MS/MS, in order to determine the slope of the terminal phase (*λ*_z_). The *λ*_z_ was calculated as the negative of the slope of the log-linear regression of the natural logarithm concentration–time curve during the terminal phase. The *λ*_z_ is an important parameter used to determine CS elimination half-life (T_1/2_), which is an index of drug persistence in the body [33]. Bioanalyses and pharmacokinetic analyses were performed as previously described [8]. The quantification of CS was based on the peak area ratio of the analyte with the internal standard. A calibration curve was used for determining the concentration of CS in all unknown samples by comparing the peak area ratio of the unknown samples to a set of standard samples of known concentration. It is important to note that a linear regression (weighted 1/concentration) produced the best fit for the concentration–detector relationship and consequently, the change of CS ionization states had a minimal effect within the analytical range used. The method precision and accuracy was well within acceptable figure of merits [34].

### Statistical analysis

Bacterial counts and CS plasma concentrations were log_10_ transformed prior to data analysis to normalize distributions. Total *E. coli* counts, ETEC: F4 counts, rectal temperature, and body weight were analyzed with repeated-measures ANOVA, with time as a within-subject factor and group as the between-subject factor. A priori contrasts were performed to compare group means at different time periods and to compare pre- and post-infection means in each treatment. For these multiple comparisons, the alpha level was adjusted downward using the Benjamini-Hochberg sequential procedure. A similar procedure was used to analyze CS plasma concentration to determine effect of ETEC: F4 oral challenge on CS intestinal absorption in pigs.

Ordinal diarrhea scores were analyzed with the Cochran-Mantel-Haenszel test at each time period.

Statistical analyses were carried out with SAS v.9.4. (Cary, N.C.). The level of statistical significance was set at *p* < 0.05 for all analyses.

## Results

During the acclimation period, none of the pigs in the two trials showed clinical signs of PWD. In trial 1, there were no deaths among pigs throughout the experiment. However, in trial 2, one pig in the challenged treated group died 2 days after the oral challenge and two pigs in the challenged untreated group died at 4 and 6 days after the challenge following presentation of a profuse diarrhea (score 4). Necropsies were not performed for dead pigs, due to the presence of advanced post-mortem bacterial invasion. However, no mortality occurred in the unchallenged groups of the two trials.

As the two trials were not performed at the same time for technical reasons, the two CS doses were only compared when the course of infection was similar for the challenged untreated groups (control groups) of the two trials. Thus, the effect of CS dose was compared between the two trials only for shedding of ETEC: F4 and total *E. coli*.

### Analysis of ETEC: F4 bacterial shedding (trial 1 and trial 2)

**Figure 1:**
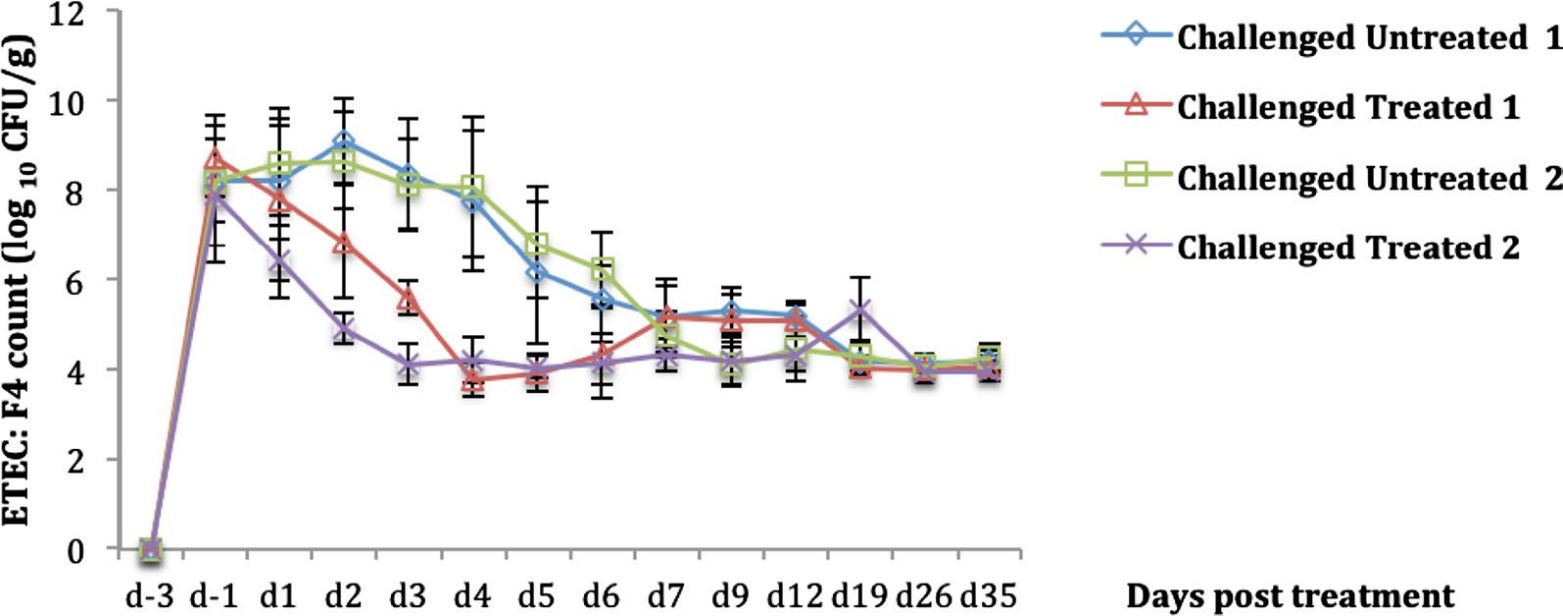
**Evolution of fecal ETEC: F4 bacterial counts (mean** **±** **standard deviation [SD]).** Challenge was performed at d-2 and treatment with colistin sulfate (CS) at a dose of 100 000 IU/kg (trial 1) or 50 000 IU/kg (trial 2) was started at d0 (36 h post challenge) and administered twice daily for a period of 5 days. In the two trials, CS treatment resulted in a significant reduction in fecal ETEC: F4 shedding between d2 and d6 (*p* < 0.0001). A significantly lower fecal excretion of ETEC: F4 was observed in trial 2 compared to trial 1 between d1 and d3 inclusive (*p* < 0.0001).

After the challenge, there was a rapid initial increase in ETEC: F4 shedding in the feces of all challenged pigs (Figure 1). There were no significant differences between the groups in the recovery of ETEC: F4 on the first day post challenge but on the following day after CS first dose administration (d1), there was a reduction in the treated group compared to the untreated group in the trial 2 (*p* < 0.0001). In both trials, CS treatment resulted in a significant reduction in fecal ETEC: F4 shedding between d2 and d6 (*p* < 0.0001), and the levels of ETEC: F4 dropped below our detection limit for most pigs between d4 and d6. However, after d6, fecal excretion of ETEC: F4 increased in the treated groups to the same level of excretion as in the untreated groups, with a significant increase at d19 in trial 2 (*p* = 0.007). However, a significant reduction in fecal excretion of ETEC: F4 was observed in trial 2 compared to trial 1 between d1 and d3, inclusively (*p* < 0.0001) (Figure 1).

In the two trials, during the acclimation period, no *E. coli* were isolated on the blood agar plates containing nalidixic acid from any fecal samples, nor from unchallenged pigs throughout the experiment.

### Analysis of shedding of total *E. coli* population (trial 1 and trial 2)

Mean total fecal *E. coli* counts of the challenged treated group and the challenged untreated group were similar on d-3 (before challenge) in each trial and increased in both challenged groups of the two trials on d-1 (24 h after challenge) (Additional file 1). However, the ETEC: F4 challenge did not significantly increase the total *E. coli* population fecal shedding.

Colistin sulfate treatment at a dose of 100 000 IU/kg (trial 1) induced a significant reduction in fecal total *E. coli* shedding between d1 and d5 in the challenged treated group compared to the challenged untreated group (*p* < 0.0001) (Additional file 1). The same therapeutic regimen (100 000 IU/kg) also resulted in a significant reduction in fecal total *E. coli* shedding between d2 and d6 in the unchallenged treated groups compared to the unchallenged untreated group (*p* < 0.0001) (Additional file 2).

Colistin sulfate treatment at a dose of 50 000 IU/kg (trial 2) induced a significant reduction in fecal total *E. coli* shedding between d1 and d6 in the challenged treated group compared to the challenged untreated group (*p* < 0.0007) (Additional file 1). This therapeutic regimen also resulted in a significant reduction in fecal total *E. coli* shedding between d2 and d5 in the unchallenged treated group compared to the unchallenged untreated group (*p* < 0.0001) (Additional file 2). However, in both trials, starting from d7 (2 days after CS cessation), fecal excretion of total *E. coli* increased in the treated groups to reach the same level of excretion as in the untreated groups (Additional files 1 and 2).

A significant reduction in fecal excretion of total *E. coli* was observed in trial 2 compared to trial 1 at d2 and d3 inclusively (*p* = 0.003 and *p* < 0.0001, respectively). Consequently, the highest reduction in total *E. coli* fecal shedding was observed in trial 2 (lower dose) between d2 and d3 (Additional files 1 and 2).

### Isolation of *E. coli* resistant to colistin sulfate

In trial 2, before the challenge period and exposure to CS at a dose of 50 000 IU/kg, fecal shedding of putative CS-resistant *E. coli* in the challenged treated group and the untreated group was very similar, as shown by the ratios of log putative CS-resistant E. coli/log total *E. coli* (Additional file 3). A low number of cultivable resident putative CS resistant *E. coli* were observed in all pigs used in this study.

Following CS administration, there was a significant decrease in the total *E. coli* population (Additional file 1). From d2 post CS treatment, the challenged treated pigs demonstrated a slight increase (15%) in the proportion of putative CS-resistant *E. coli* compared with the challenged untreated pigs. This difference was observed throughout CS administration, being significant between d3 and d5 (*p* < 0.0005) and gradually diminishing from the first day (day 6) of CS discontinuation (Additional file 3).

Among 80 putative CS resistant *E. coli* isolates on MacConkey plates, 72 were identified as *E. coli* by biochemical analyses, only one isolate being identified as ETEC: F4 by multiplex PCR. No putative CS resistant colonies were isolated on blood agar plates containing nalidixic acid.

Among 72 putative CS resistant *E. coli* isolates, 9 (8 in the challenged treated group and one in the challenged untreated group) were confirmed resistant to CS with an MIC >2 μg/mL (Table 1).

**Table 1:** **Distribution** **of minimal inhibitory concentrations of porcine CS resistant** ***E. coli*** **isolates in trial 2**

			Colistin sulfate MIC values (μg/mL)									
Isolates	Time	Groups	0.06	0.12	0.25	0.5	1	2	4	8	16	32
M4A3	D3	CT								**+**		
M4B3	D3	CT								**+**		
M4C3	D3	CT								**+**		
M4D3	D3	CT								**+**		
M6A11	D11	CT									**+**	
M6C11	D11	CT								**+**		
M6B11	D11	CT								**+**		
L10A4^a^	D4	CU								**+**		
L1B1	D1	CT							**+**			

D3 = 3 days post CS treatment; D11 = 11 days post CS treatment; D4 = 4 days post challenge; D1 = 1 day post CS treatment.

The isolates with MIC values higher than resistance breakpoint (MIC > 2 μg/mL) as described by Li et al. [45] were considered resistant.

MIC of ECL8559A <0.06 μg/mL.

CU: challenged untreated; CT: challenged treated.

^a^Isolate confirmed ETEC: F4 by multiplex PCR.

The CS resistant ETEC: F4 isolate, probably originating from the challenge strain as it was confirmed by multiplex PCR, demonstrated an MIC of 8 μg/mL, as compared to <0.06 μg/mL for the challenge strain (ECL8559A). This ETEC: F4 isolate was found in the challenged untreated group 4 days after the oral challenge (Table 1).

### Analysis of health status and growth performance

Prior to bacterial challenge, no pig in either trial showed any indication of severe diarrhea or loose stools. None of the unchallenged pigs in the two trials showed any illness or diarrhea during the experiment.

**Figure 2:**
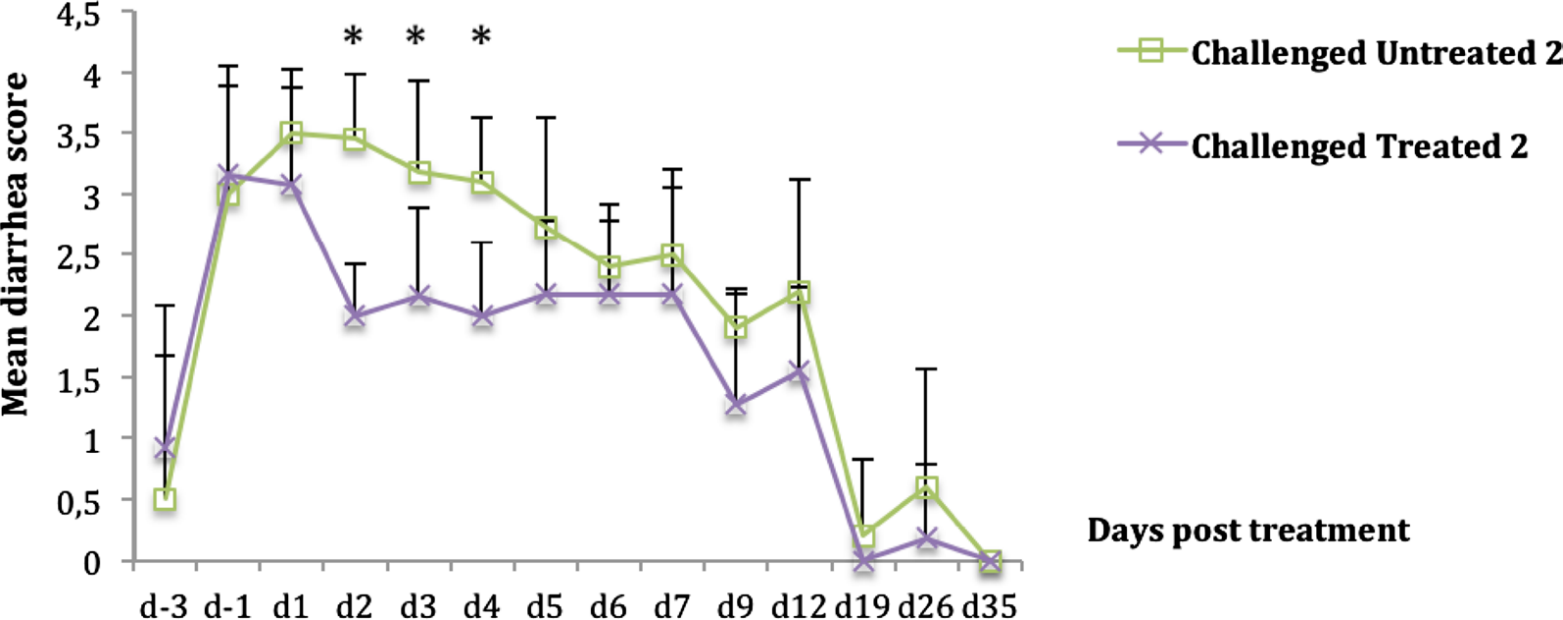
**Mean diarrhea score (±standard deviation [SD]) of weaned pigs challenged with ETEC: F4.** Challenge was performed at d-2 and treatment with colistin sulfate (CS) at the dose of 50 000 IU/kg (trial 2) was started at d0 (36 h post challenge) and administered twice daily for a period of 5 days. Treatment with oral CS resulted in a statistically significant reduction in the diarrhea score of the challenged treated group compared to the challenged untreated group (*p* < 0.0001) between d2 and d4. Mean diarrhea score = sum of daily diarrhea score/number of animals. *: *p* < 0.0001.

Following challenge, all challenged pigs in the two trials showed high diarrhea scores with no statistically significant difference between treated and untreated groups (Figure 2; Additional file 4). After 2 days of CS administration (d2), diarrhea scores were significantly decreased in the challenged treated compared to the challenged untreated groups, and shown in Figure 2 for trial 2 (*p* < 0.0001). The decrease was also observed at d3 and d4 in the two trials (Figure 2; Additional file 4). From d5 (6 days post challenge), diarrhea scores in the challenged untreated groups of both trials decreased and no statistically significant difference in the diarrhea scores between challenged untreated and challenged treated groups was observed in either trial.

Some challenged pigs in both trials developed hypothermia, several days post challenge, occasionally followed by death.

**Figure 3:**
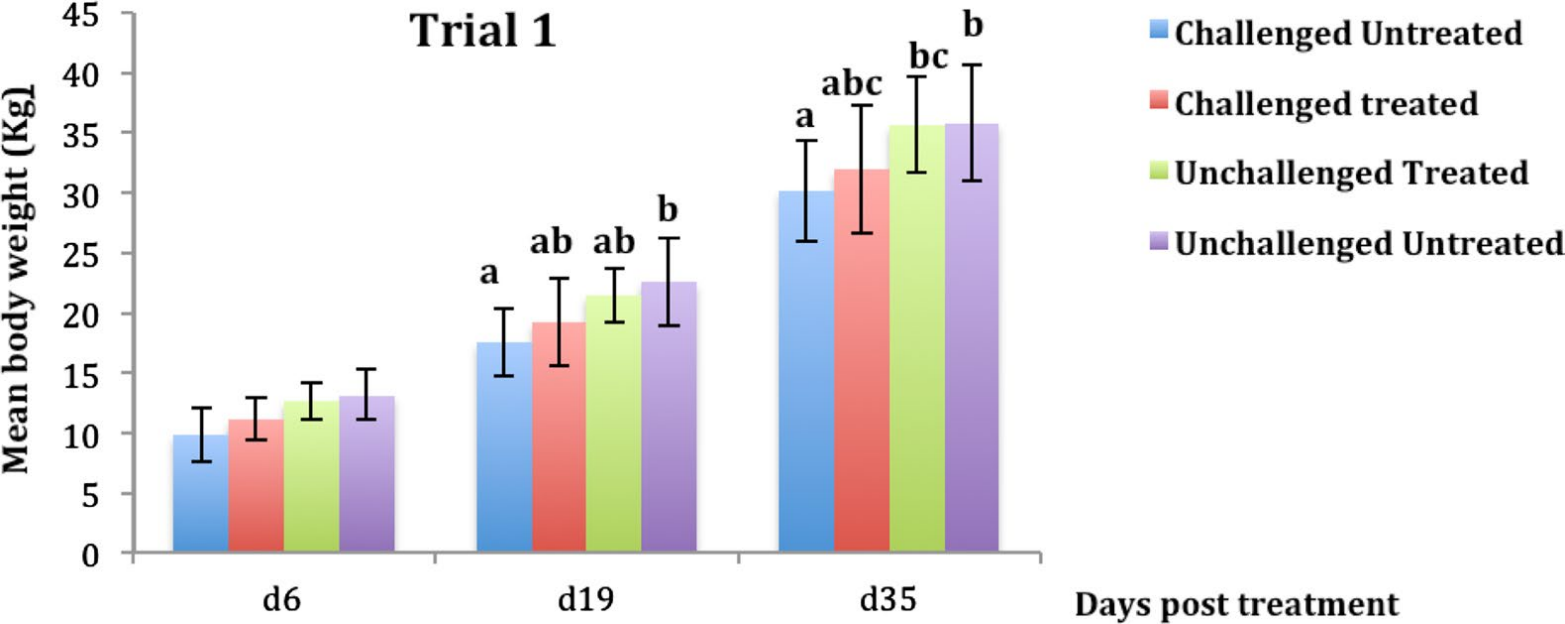
**Evolution of body weight in pigs receiving colistin sulfate (CS)** **orally at a dose of 100 000** **IU/kg (mean** **±** **standard deviation [SD]).** Challenge was performed at d-2 and treatment with colistin sulfate (CS) at the dose of 100 000 IU/kg. For each sampling time, means with different letters on a given day are statistically different. At d6 there was no significant difference between groups.

**Figure 4:**
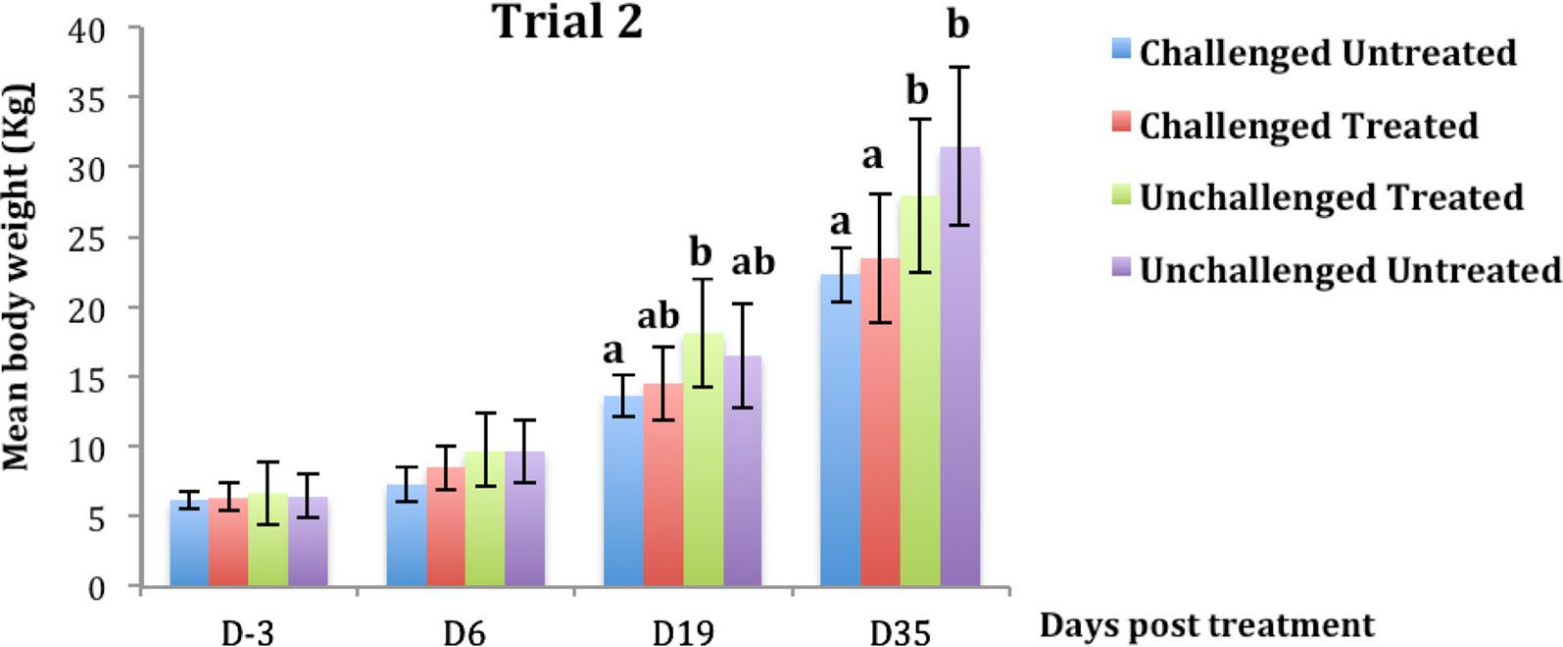
**Evolution of body weight in pigs receiving colistin sulfate (CS)** **orally at a dose of 50 000** **IU/kg (mean** **±** **standard deviation [SD]).** Challenge was performed at d-2 and treatment with colistin sulfate (CS) at the dose of 50 000 IU/kg. For each sampling time, means with different letters on a given day are statistically different. At d-3 and d-6 there was no significant difference between groups.

The body weight of the pigs in trial 2 in the pre-challenge period did not differ among the four groups (*p* > 0.71). Following oral challenge with ETEC: F4 and CS treatment discontinuation (d6), no difference was detected in the body weight of all pigs in both trials (Figures 3 and 4) with *p* > 0.05 and *p* > 0.07 for trials 1 and 2 respectively. After 2 weeks of CS treatment discontinuation (d19), a significantly higher body weight was observed in trial 1 for the unchallenged untreated (control) compared to the challenged untreated group (*p* < 0.001) (Figure 3). However, in trial 2 for the same time (d19), the unchallenged treated group presented a higher mean weight compared to the challenged untreated group (*p* < 0.001) (Figure 4). After 30 days of CS treatment discontinuation (d35) in both trials, the unchallenged treated and the control groups presented a higher mean weight compared to the challenged untreated groups (Figures 3 and 4). In addition, in trial 2 at d35, the unchallenged treated group and the control group presented a higher mean weight compared to the challenged treated group, with *p* < 0.0001.

Overall, the ETEC: F4 challenge resulted in decreased growth rate of the challenged groups in both trials and treatment with CS at the doses used in this study did not affect this decreased growth rate.

### Quantification of plasma concentration of colistin sulfate and pharmacokinetic analysis

**Figure 5:**
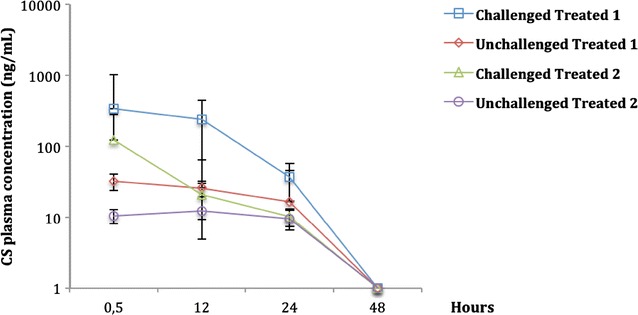
**Evolution of plasma CS concentrations over time in pigs challenged with an ETEC: F4 strain and receiving colistin sulfate (CS) orally (mean** **±** **standard deviation [SD]).** Colistin sulfate concentrations were obtained by HPLC–MS/MS after 0.5, 12, 24 and 48 h of CS treatment discontinuation at a therapy regimen of 100 000 IU/kg (trial 1) or 50 000 IU/kg (trial 2). In trial 1, at 0.5, 12 and 24 h, CS concentrations were statistically higher in the challenged treated group compared to the unchallenged treated group with *p* < 0.001, *p* < 0.0001 and *p* < 0.001 respectively. In trial 2, at 0.5 and 12 h, CS concentration was statistically higher in the challenged treated group compared to the unchallenged treated group with *p* < 0.001 and *p* = 0.04 respectively (*n* = 8 per group).

In order to determine whether ETEC: F4 challenge affects CS intestinal absorption, an HPLC–MS/MS was used for CS quantification in pig plasma. The lower limit of quantitation (LLOQ) of our method was 1 ng/mL of plasma. The pharmacokinetic analyses were performed using a non-compartmental model. In both trials, CS plasma concentrations were detected in all treated groups (challenged or not), although they were higher in challenged treated groups compared to the unchallenged treated groups for all sampling times (Figure 5).

In the challenged treated groups, the mean of *C*_max_ (± SD) (the observed maximum plasma concentration of CS) was 338.3 (±676.37) and 122.3 (±161.97) ng/mL at 0.5 h post CS treatment discontinuation in trials 1 and 2 respectively (Figure 5). In trial 1, at 0.5, 12 and 24 h after CS treatment discontinuation, CS plasma concentrations were statistically higher in the challenged treated group compared to the unchallenged treated group with *p* < 0.001, *p* < 0.0001, and *p* < 0.001 respectively. The same finding was observed in trial 2, the CS plasma concentrations were higher in challenged treated compared to the unchallenged treated group at 0.5 h (*p* < 0.001), and at 12 h (*p* = 0.04). Thus, ETEC: F4 oral challenge exacerbated the intestinal absorption of CS in challenged compared to unchallenged weaned pigs. In both trials, at 48 h following the last CS administration, plasma concentrations were below the LLOQ of our method. We were not able to determine the *λ*_z_ and T_1/2_ of CS following its oral administration even in challenged treated pigs. Based on our sampling plan it was not possible to characterize the CS elimination phase and make a linear regression of the last CS plasma concentrations.

## Discussion

The aim of the present study was to evaluate the impact of CS on the *E. coli* populations and pig health status in experimental *E. coli*-induced diarrhea in weaned pigs. We also studied the impact of ETEC: F4 oral challenge on CS intestinal absorption level in pigs using a highly sensitivity analytical method (HPLC–MS/MS).

The duration of the experiment was 35 days in each trial, to cover the withdrawal period of 30 days applied in Canada for CS in pig farms. Indeed, in the absence of scientific explanation for the difference in the withdrawal period for CS oral formulations in pigs between countries [6], veterinarians use this long time period of 30 days as a safety measure for consumer protection against potential CS chemical residues in pig meat.

We used two doses of CS in our study in order to more closely reflect farm practices. In fact, the lower dose (50 000 IU/kg) is the recommended therapeutic dose in pigs, whereas the higher dose (100 000 IU/kg) was used to take into consideration a more realistic portrait of CS use on pig farms, where this antibiotic is often overdosed [23], and the social rank and heterogeneity observed among pigs in the same pens which may increase antimicrobial consumption for some pigs [35].

In the current study, maximum ETEC: F4 shedding and diarrhea scores were observed one-day post challenge. This result is consistent with other experimental studies in which a higher frequency of watery diarrhea was observed after the first day of the ETEC: F4 oral challenge [36, 37].

In our study, regardless of the dose, CS treatment led to a decrease of nearly 4 log cfu/g in fecal shedding of ETEC: F4 and total *E. coli,* but only during the treatment period. This finding corroborates the study of Torrallardona et al. who showed that the use of CS at a dose of 300 mg/kg of diet in the treatment of weanling pigs challenged with *E. coli* K99 for a period of 7 or 14 days was associated with a reduction of the number of *E. coli* in both ileal and cecal digesta by 5.30 and 4.38 log cfu/g, respectively [38]. In our study, the effect of CS on the decrease of ETEC: F4 and total *E. coli* population was greater with the low dose of CS (50 000 IU/kg) used in trial 2. This finding is in disagreement with the known pharmacodynamics (PD) of CS as an antibiotic that exhibits its bactericidal activity in a concentration-dependent manner in vitro [22]. However, Lin et al. reported that CS bioavailability after an intramuscular (IM) administration in pigs, was inversely proportional with the administered CS doses, with a systemic bioavailability of 95.94 and 88.45% for 2.5 and 5 mg/kg bw respectively [39].

In the current study, no difference was noted between low and high CS doses given to pigs, regarding *E. coli* recovery and on health status. Nevertheless, it would have been interesting to quantify colistin in pig gut, to link the microbiological effects determined to the real CS concentrations in intestinal segments. However, for logistic reasons associated with the design of the experiment and due to the low number of pigs in each group, it was not possible to sacrifice animals to recover the digestive contents, in this study.

In the present study, after CS treatment discontinuation in the two trials, there was no difference in fecal shedding of ETEC: F4, total *E. coli* population, and diarrhea scores between challenged treated and challenged untreated groups. However, it should be noted that our experiment was carried out in controlled conditions, and that the outcome of CS treatment may differ during natural infections in farm conditions associated with specific factors such as livestock management, presence of other infections in the farm, feed additives, vaccination or other factors.

In our study, 12.5% of *E. coli* isolates originating from growth on MacConkey agar plates with 2 μg/mL of CS were confirmed resistant to colistin, most (8/9) following the treatment with CS at 50 000 IU/kg, suggesting a CS selection pressure on *E. coli*. Our results corroborate those of Boyen et al. who determined that approximately 10% of the 157 investigated porcine *E. coli* isolates from sick pigs showed resistance to colistin [16]. However, it is not clear whether sampled animals were treated with colistin in this study. On the other hand, the MICs of CS *E. coli* resistant isolates determined in our study were in the same range as those of resistant *E. coli* isolated from sick pigs in farm conditions [13, 16].

In the present study, the CS resistance was observed in 3 *E. coli* isolates even 6 days after CS treatment discontinuation, and in an isolate confirmed ETEC: F4 in the challenged untreated group 4 days after the oral challenge. Further investigations are ongoing to explain if this CS resistance is associated with chromosomal mutations or a plasmid resistance gene, and to determine the origin of the higher MIC observed for the ETEC: F4 isolate compared to the challenge strain by determining of its natural mutation rate.

Although we observed a lower proportion of CS *E. coli* resistant isolates than reported by other authors [12, 40], it is premature to confirm that the use of this CS regimen in pigs is associated with a low resistance among *E. coli*. It would be interesting to determine in a future study the effect of CS in a mass treatment (drinking water or in feed) on CS resistance in *E. coli* in pig farm conditions and following a repetitive CS treatment.

In our study, the MacConkey agar plates supplemented with 2 μg/mL of CS overestimated the number of resistant *E. coli* as only a small percentage of the *E. coli* recovered from these MacConkey agar plates could be confirmed resistant to CS by MIC determination using Mueller–Hinton broth media. This finding may be due to the culture media change between the two experiments as well as the difference in the matrix used: fecal material for MacConkey agar plates versus pure culture for Mueller–Hinton. In our study, the use of the MacConkey supplemented with 2 μg/mL of CS served mostly as a screening step for reducing the numbers of isolates potentially sensitive to CS and thus limiting the number of isolates to be tested on Mueller–Hinton for CS resistance confirmation. Our study underlines the importance of confirming putative CS isolates on Mueller–Hinton CMI determination when non-standardized culture media are used for assessing the resistance levels of a given bacterial population.

In the present study, a growth retardation was observed in surviving animals of the challenged groups compared with the unchallenged groups in the two trials. This finding corroborates the study of Bontempo et al. who showed that *E. coli* challenge significantly impairs performance, resulting in a reduction of average daily gain for pigs [41]. Colistin sulfate treatment in the two trials did not prevent pig weight losses in challenged treated compared to challenged untreated pigs. In addition, we have not noticed a difference in pig body weight between unchallenged treated and unchallenged untreated groups in both trials. To the best of our knowledge, our study is the first to report these results following an oral CS administration at 50 000 or 100 000 IU/kg bw in pigs. Nevertheless, it will be interesting to investigate in a long-term field trial with more pigs and in field conditions the effect of CS therapeutic regimen on pig weight loss prevention in the post-weaning period.

In our study, ETEC: F4 oral challenge increased the passage of CS from the intestine to the blood in the challenged pigs compared to the unchallenged weaned pigs in the two trials. Several studies have shown that administration of bacterial lipopolysaccharide (LPS) results in the production and release of TNF-α and IL-1; these pro-inflammatory cytokines increased epithelial tight junction permeability in vitro in Caco-2 cells [42]. In another study, it was demonstrated that IL-1, activated endothelial cells (EC) to induce vascular leakage via loss of vascular endothelial (VE)-cadherin [43]. The role of LPS release by the challenge ETEC: F4 strain in increasing pig intestinal tight junction permeability and pro-inflammatory cytokine production needs to be confirmed in a future study.

Our results demonstrated that *E. coli* intestinal infection in weaned pigs with clinical PWD symptoms, resulted in increased of CS intestinal absorption. This finding should be taken into consideration when determining CS withdrawal time, bearing in mind that withdrawal times are mostly determined in healthy animals [44], even though antibiotics are currently used to treat clinically sick pigs.

In conclusion, this is the first report on the use of CS for the treatment of experimental *E. coli*-induced diarrhea in weaned pigs. In our study, we determined that under controlled conditions in pigs, CS reduced ETEC: F4 and *E. coli* fecal shedding and diarrhea scores during treatment period. However, CS treatment did not prevent pig weight losses due to the diarrhea and exerted a slight selection pressure on the CS resistant *E. coli* commensal population. In addition, we demonstrated that oral challenge of pigs using an ETEC: F4 strain increased passage of CS from the intestine to the blood. This observation should be taken into consideration when determining the oral CS withdrawal time in pigs.

A longer duration field trial investigation is recommended to better understand the relationship between CS effectiveness and CS bacterial resistance following the use of oral CS in PWD control in commercial farm conditions and lead to a prudent use of antimicrobials in swine medicine.

## Additional files

10.1186/s13567-016-0344-y **Evolution of fecal total** ***E. coli*** **counts (mean** **±** **standard deviation [SD]) in challenged groups**. Challenge was performed at d-2 and treatment with colistin sulfate (CS) at the dose of 100 000 IU/kg (trial 1) or 50 000 IU/kg (trial 2) was started at d0 (36 h post challenge) and administered twice daily for a period of 5 days. CS treatment resulted in a significant reduction in fecal total *E. coli* shedding between d2 and d5 in trial 1 and between d1 and d6 in trial 2 in the challenged treated group compared to the challenged untreated group (*p* < 0.0001).
10.1186/s13567-016-0344-y **Evolution of fecal total** ***E. coli*** **counts (mean** **±** **standard deviation [SD]) in unchallenged groups.** Treatment with colistin sulfate (CS) at a dose of 100 000 IU/kg (trial 1) or 50 000 IU/kg (trial 2) was started at d0 (36 h post challenge) and administered twice daily for a period of 5 days. CS treatment resulted in a significant reduction in fecal total *E. coli* shedding between d2 and d6 in trial 1 and between d2 and d4 in trial 2 in the unchallenged treated groups compared to the unchallenged untreated (control) groups (*p* < 0.0001).
10.1186/s13567-016-0344-y **Evolution of fecal ratio of putative CS-resistant** ***E. coli*** **/total** ***E. coli*** **counts (mean** **±** **standard deviation [SD])**. Challenge was performed at d-2 and colistin sulfate (CS) was administered at the dose of 50 000 IU/kg twice daily for 5 days, starting at d0 (36 h post challenge). CS treatment induced a significant increase in fecal putative CS-resistant *E. coli* (selective pressure) shedding between d3 and d5 in the challenged treated group compared to the challenged untreated group *: *p* < 0.0001. ******: *p* < 0.001.
10.1186/s13567-016-0344-y **Mean diarrhea score (±standard deviation [SD]) of weaned pigs challenged with ETEC: F4**. Challenge was performed at d-2 and treatment with colistin sulfate (CS) at a dose of 100 000 IU/kg (trial 1) was started at d0 (36 h post challenge) and administered twice daily for a period of 5 days. Treatment with oral CS had led to a statistically significant reduction in the diarrhea score of the challenged treated group compared to the challenged untreated group (*p* < 0.0001) on d2 and d4. Mean diarrhea score = sum of daily diarrhea score/number of animals (*n* = 12 per group). *: *p* < 0.0001.
