# Supplementary figures and images for: In vivo therapeutic efficacy and pharmacokinetics of colistin sulfate in an experimental model of enterotoxigenic Escherichia coli infection in weaned pigs
Source: Vet Res. 2016 May 27;47:58. doi: 10.1186/s13567-016-0344-y (PMC4884413; doi:10.1186/s13567-016-0344-y)

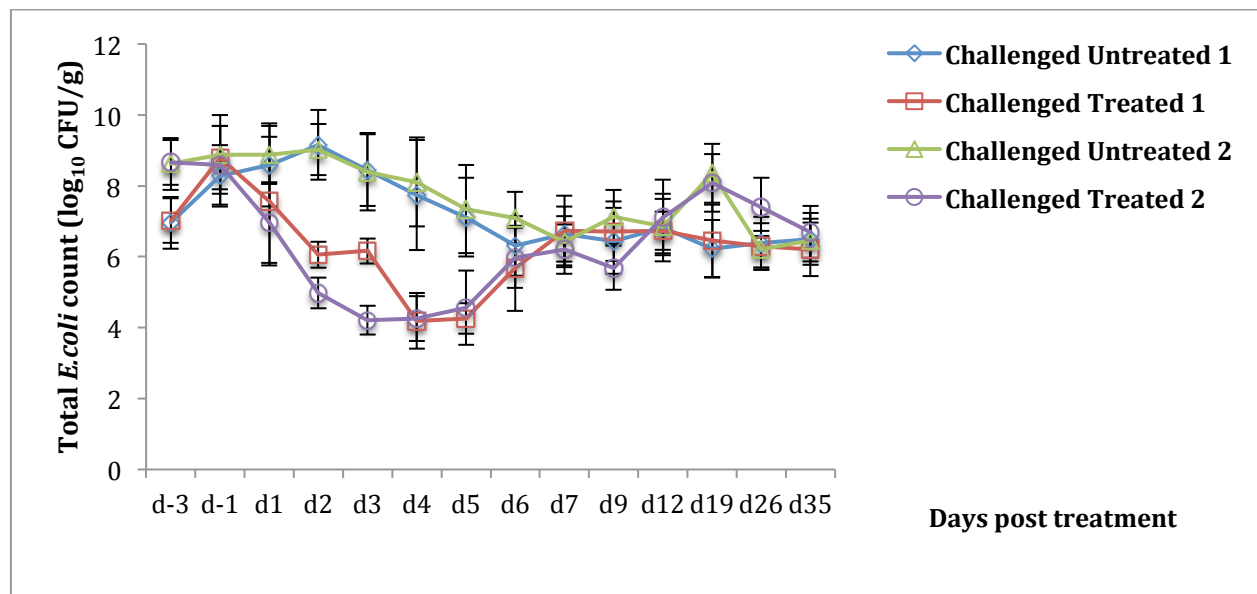

Supplement: Supplementary file 1 — 10.1186/s13567-016-0344-y Evolution of fecal total E. coli counts (mean ± standard deviation [SD]) in challenged groups. Challenge was performed at d-2 and treatment with colistin sulfate (CS) at the dose of 100 000 IU/kg (trial 1) or 50 000 IU/kg (trial 2) was started at d0 (36 h post challenge) and administered twice daily for a period of 5 days. CS treatment resulted in a significant reduction in fecal total E. coli shedding between d2 and d5 in trial 1 and between d1 and d6 in trial 2 in the challenged treated group compared to the challenged untreated group (p < 0.0001). [file 13567_2016_344_MOESM1_ESM.pdf]

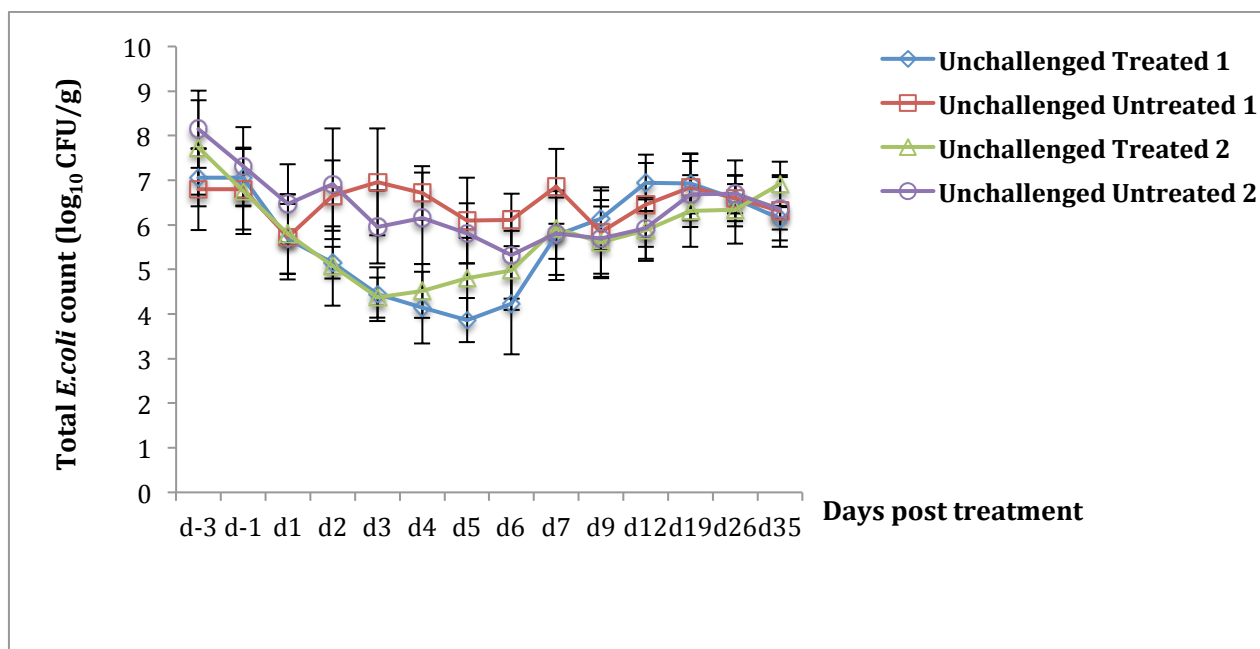

Supplement: Supplementary file 2 — 10.1186/s13567-016-0344-y Evolution of fecal total E. coli counts (mean ± standard deviation [SD]) in unchallenged groups. Treatment with colistin sulfate (CS) at a dose of 100 000 IU/kg (trial 1) or 50 000 IU/kg (trial 2) was started at d0 (36 h post challenge) and administered twice daily for a period of 5 days. CS treatment resulted in a significant reduction in fecal total E. coli shedding between d2 and d6 in trial 1 and between d2 and d4 in trial 2 in the unchallenged treated groups compared to the unchallenged untreated (control) groups (p < 0.0001). [file 13567_2016_344_MOESM2_ESM.pdf]

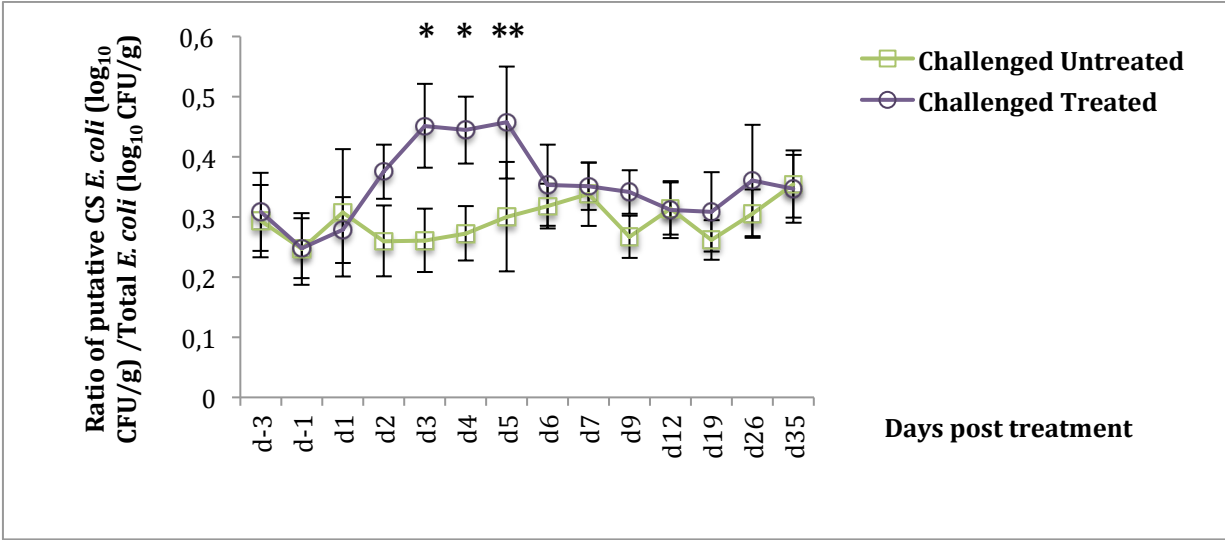

Supplement: Supplementary file 3 — 10.1186/s13567-016-0344-y Evolution of fecal ratio of putative CS-resistant E. coli /total E. coli counts (mean ± standard deviation [SD]). Challenge was performed at d-2 and colistin sulfate (CS) was administered at the dose of 50 000 IU/kg twice daily for 5 days, starting at d0 (36 h post challenge). CS treatment induced a significant increase in fecal putative CS-resistant E. coli (selective pressure) shedding between d3 and d5 in the challenged treated group compared to the challenged untreated group *: p < 0.0001. **: p < 0.001. [file 13567_2016_344_MOESM3_ESM.pdf]

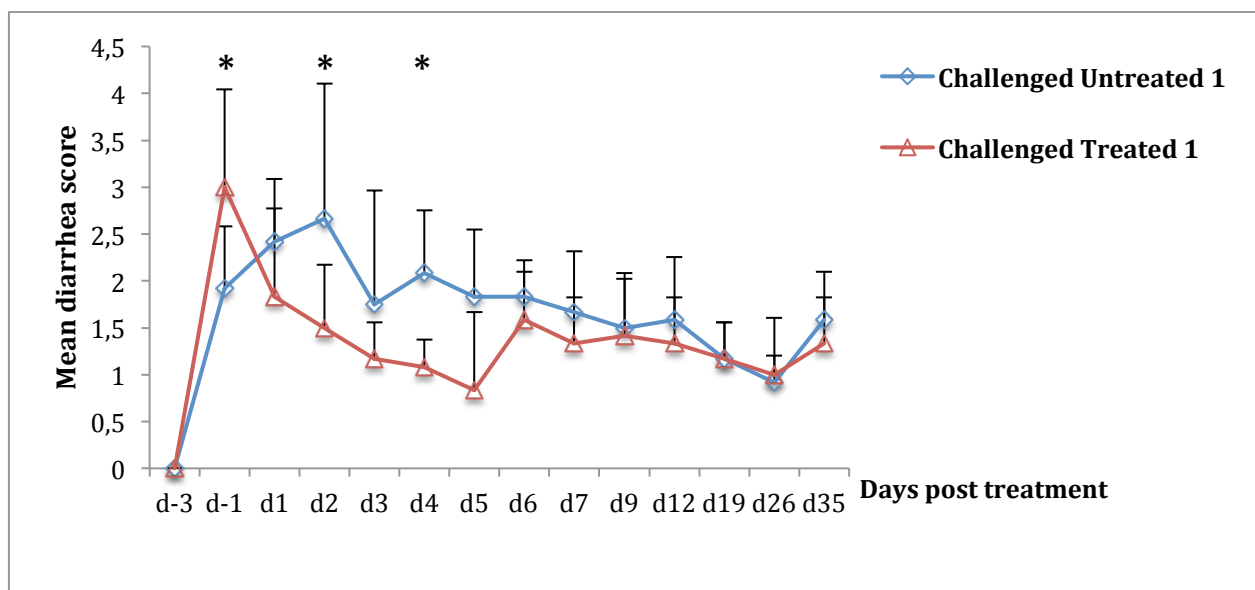

Supplement: Supplementary file 4 — 10.1186/s13567-016-0344-y Mean diarrhea score (±standard deviation [SD]) of weaned pigs challenged with ETEC: F4. Challenge was performed at d-2 and treatment with colistin sulfate (CS) at a dose of 100 000 IU/kg (trial 1) was started at d0 (36 h post challenge) and administered twice daily for a period of 5 days. Treatment with oral CS had led to a statistically significant reduction in the diarrhea score of the challenged treated group compared to the challenged untreated group (p < 0.0001) on d2 and d4. Mean diarrhea score = sum of daily diarrhea score/number of animals (n = 12 per group). *: p < 0.0001. [file 13567_2016_344_MOESM4_ESM.pdf]
